# Comprehensive Analysis of RNA Expression Correlations between Biofluids and Human Tissues

**DOI:** 10.3390/genes12060935

**Published:** 2021-06-18

**Authors:** Ruya Sun, Chunmei Cui, Yuan Zhou, Qinghua Cui

**Affiliations:** Key Laboratory of Molecular Cardiovascular Sciences of the Ministry of Education, Center for Non-Coding RNA Medicine, Department of Biomedical Informatics, School of Basic Medical Sciences, Peking University Health Science Center, Beijing 100191, China; sunruya@pku.edu.cn (R.S.); ccm328@bjmu.edu.cn (C.C.); zhouyuanbioinfo@hsc.pku.edu.cn (Y.Z.)

**Keywords:** biofluid, tissue, mRNA, lncRNA, biomarker

## Abstract

In recent years, biofluid has been considered a promising source of non-invasive biomarkers for health monitoring and disease diagnosis. However, the expression consistency between biofluid and human tissue, which is fundamental to RNA biomarker development, has not been fully evaluated. In this study, we collected expression profiles across 53 human tissues and five main biofluid types. Utilizing the above dataset, we uncovered a globally positive correlation pattern between various biofluids (including blood, urine, bile, saliva and stool) and human tissues. However, significantly varied biofluid–tissue similarity levels and tendencies were observed between mRNA and lncRNA. Moreover, a higher correlation was found between biofluid types and their functionally related and anatomically closer tissues. In particular, a highly specific correlation was discovered between urine and the prostate. The biological sex of the donor was also proved to be an important influencing factor in biofluid–tissue correlation. Moreover, genes enriched in basic biological processes were found to display low variability across biofluid types, while genes enriched in catabolism-associated pathways were identified as highly variable.

## 1. Introduction

Currently, with the rapid development of the medical industry, the early detection of diseases, as well as the monitoring of health status, become increasingly applicable and urgently needed [1]. Traditionally, disease diagnosis is completed through clinical tests of relevant tissues, which is direct but usually invasive. Multiple tissues participate in the production, secretion and transportation procedures of biofluids [2]. Human biofluids refer to biological fluids secreted or excreted from inside body, including but not limited to blood, urine, sweat, and tears [3]. Compared to tissue samples, biofluids are more easily obtained clinically, as they can usually be acquired via a needle or swab. Considering the advantages of a consistently rich source and their ability to be acquired by non-invasive means, biofluids are believed to be another important source of biomarkers for disease diagnosis and monitoring [4]. In fact, multiple biofluids have already been proven to be promising for clinical practice [5], for example, in the detection of Huntington’s Disease (HD) [6], colorectal cancer (CRC) [7], traumatic brain injury (TBI) [8], etc.

To better understand and guide the clinical application of biofluids, we still need to ascertain, as the end-point product, to what degree the composition and abundance of gene expression products agrees between human tissues and biofluids. Here, we focused on RNA products and compared the expression profiles between various human tissues and a range of human biofluids. We analyzed the consistency and divergence between healthy human tissue and biofluids, and factors that might influence their correlation. Our group had already confirmed the extensive correlation of microRNA expression between tissues and biofluids [9]. In this study, we further extended the scope to messenger RNAs (mRNAs) and long non-coding RNAs (lncRNAs), which are also the main classes of the human transcriptome. A similar biofluid–tissue correlation pattern was identified for the whole transcriptome, mRNA and lncRNA. In order to further our understanding, we identified the top 500 lowly variable genes (LVGs) and top 500 highly variable genes (HVGs) across the biofluid transcriptomes. To figure out what role was played by the constant and hypervariable component of the biofluid transcriptome, a corresponding functional enrichment analysis was also separately performed.

## 2. Methods

### 2.1. Data Collection

The expression profiles of 53 healthy human tissues were obtained from the GTEx dataset (https://www.gtexportal.org/home/datasets, release v7, accessed on January 2021) [10]. The transcriptome profiles of 5 main biofluid types (blood, urine, saliva, bile and stool) were separately collected from 4 GEO datasets (https://www.ncbi.nlm.nih.gov/geo/, accessed on January 2021) and the exRNA Atlas database (http://exrna-atlas.org/, accessed on January 2021) [11]. The data sources for the biofluid transcriptome profiles are shown in Table S1. Considering that the GTEx dataset only collected healthy donor genome profiles, the transcriptome profiles of biofluids sampled from individuals with a disease status were not employed in the subsequent study.

### 2.2. Data Preprocess

To reduce any technical bias brought about by gene length and sequence depth, transcripts per million (TPM) normalization was first performed on the RNA-seq expression profiles included in this study. For this purpose, the human gene annotation file downloaded from GENCODE [12] was used as a reference. The expression matrices of all of the collected biofluids were merged into a single matrix, in which only intersecting genes were kept. Furthermore, to improve the quality of the analysis, genes expressed in less than 25% of biofluid samples were excluded from the integration matrix. To further mitigate the batch effect associated with the data sources, the ‘removeBatchEffect’ function of the limma R package (v3.40.6) was run. Taking GRCh38 release 13 genome (GRCh38.p13) as the reference, mRNA and lncRNA transcriptomes were next filtered out from the above-integrated whole transcriptome matrix, resulting in expression profiles for 20,845 unique ENSGs, covering 15,934 mRNAs and 4555 lncRNAs for later use.

### 2.3. Statistical Analysis

Statistical analysis was performed using R 3.6.1 (https://www.R-project.org/, accessed on January 2021) [13]. The correlation between human tissue and the biofluid transcriptome was evaluated by Spearman’s correlation (‘cor.test’ function, stats R package, v3.6.3) and defined as the biofluid–tissue similarity index (BTSI). The expression of mRNAs and lncRNAs in the biofluid and the tissue were correspondingly defined as BF-mRNAs, BF-lncRNAs, T-mRNAs and T-lncRNAs.

To enable an overview of BTSI between the various biofluids and human tissues, the average expression value of the genes in all of the GTEx samples, and the samples of each biofluid type, were calculated and used for BTSI calculation. To investigate the tendency for biofluid–tissue correlation, the average expression value of the genes in the samples of each GTEx tissue type and each biofluid type was calculated for the BTSI calculation. Furthermore, to verify the intended correlation between specific biofluid–tissue pairs, BTSI values between biofluid–tissue samples and those between biofluid-randomized GTEx samples (*A* = 1000) were compared using a *t*-test, repeated 10 times. Only significant biofluid–tissue correlations (Spearman’s correlation, *p*-value < 0.05) with a BTSI higher than the randomized biofluid–GTEx samples, as well as with a *p*-value < 0.05 in all of the above 10 repetitions (*t*-test), were labeled as intended correlations.

To find out the factors (sex, gene biotype) that might affect the biofluid–tissue correlation, a *t*-test was performed. Considering the GTEx provided us with the sex information of all sample donors. At the same time, a part of the biofluid samples provided the sex of donors and included samples from both sexes (urine exRNA, stool seRNA). The BTSI between the biofluid and the tissue sampled from both male, both female and donors of each different sex were compared using *t*-test.

In each biofluid sample, the number of genes with an expression level larger than zero (non-zero gene) was also calculated. To study the effect of the non-zero gene number on the BTSI level, linear regression was performed (‘lm’ function, VGAM R package, v1.1-5). Considering most transcriptomes of the biofluids were profiled by RNA-seq and were all processed via TPM normalization, the array profile of stool-derived eukaryotic RNA (seRNA) was excluded from this part of the analysis. 

### 2.4. Decomposition of Tissue Component in Biofluid

The deconvolution tool dtangle [14] (R package, v2.0.9) was used to infer the tissue derivation of RNAs in different biofluids. The transcriptomes of 53 human tissues obtained from the GTEx dataset were used as a reference panel to deconvolute the tissue components of various biofluids (i.e., the estimated contribution of each tissue to the transcriptome of various biofluids). To further verity the dtangle deconvolution result, the cell type enrichment tool xCell [15] (R package, v1.1.0) was also applied to infer the proportion of cell types in the biofluid samples. 

### 2.5. Functional Enrichment Analysis of Stable and Variable Components of Biofluid Genome

To identify LVGs and HVGs among biofluids, the ‘FindVariableFeatures’ function provided by the Seurat R package (v3.2.2) was applied. Generally, the variance of each gene was calculated based on local polynomial regression (LOESS). Then, the genes with the top 500 highest and lowest variance values were separately defined as the top 500 HLVs and LVGs. To explore the roles played by the top 500 HVGs and LVGs, gene ontology (GO) enrichment analysis was performed via the ‘enrichGO’ function (clusterProfiler R package, v3.12.0). Using the ‘org.Hs.eg.db’ OrgDb subject for background annotation, the corresponding enriched GO terms were obtained based on a gene ontology over-representation test.

## 3. Results

### 3.1. Biofluids and Tissues Exhibit Widely Positive Correlations

**Figure 1:**
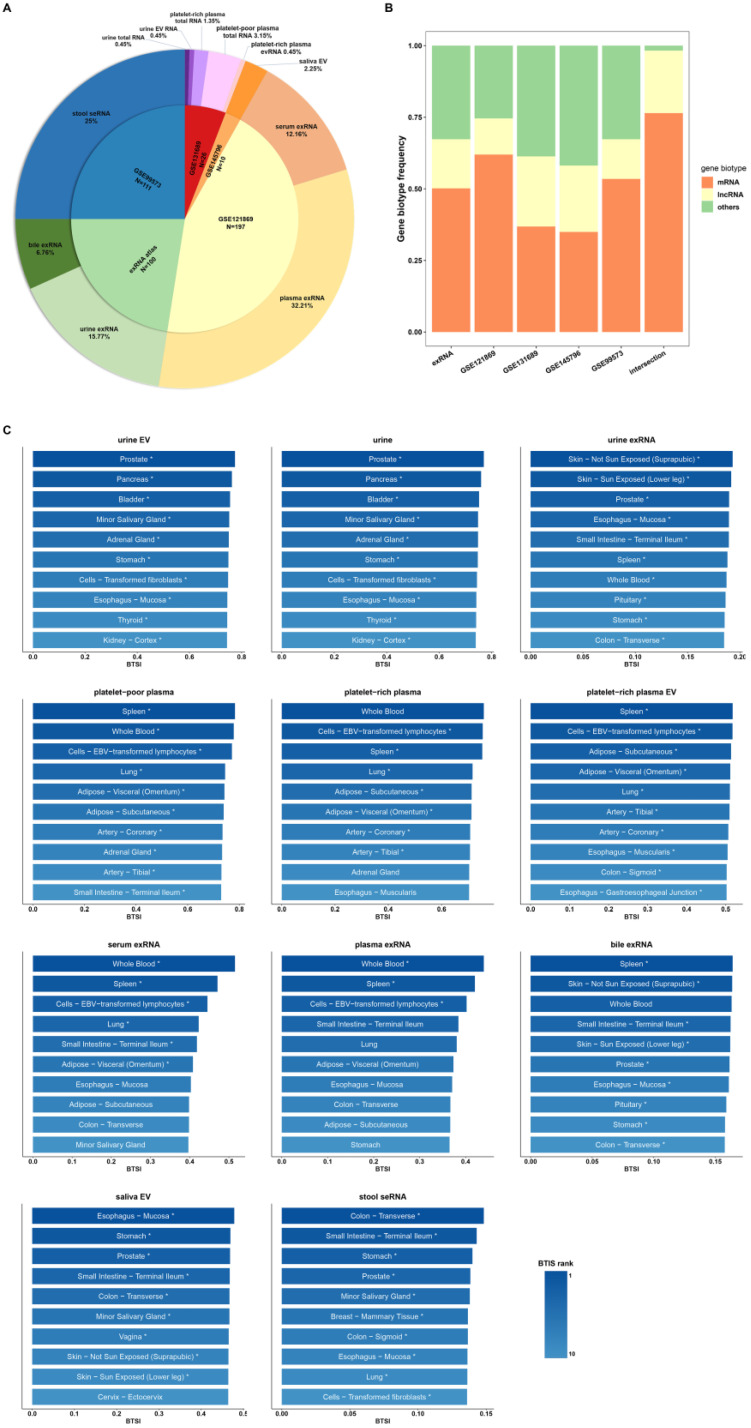
Overview of biofluid–tissue expression correlations. (**A**) Data source of collected biofluid genome profiles. The number of samples collected from each data source and the proportion of samples in the whole sample pool (*n* = 444) are labeled in the inner and outer pies separately. (**B**) Frequency of mRNA and lncRNA in transcriptome profile collected from different data sources and final intersection matrix used for analysis. The color of the bars corresponds to different gene biotypes. (**C**) The bar plot shows the tissues of the top 10 highest BTSI value for each biofluid type from whole transcriptome view. Only tissues significantly correlated with biofluid (Spearman’s correlation, *p*-value < 0.05) and presented higher correlation with biofluid samples than randomized GTEx samples (*n* = 1000; *t*-test, 10 times repeat, *p*-value < 0.05) are labeled using ‘*’. The color of each bar represents the rank of the tissue BTSI value for the corresponding biofluid.

A wide range of biofluid transcriptomic expression profiles were collected (Figure 1A,B). In general, the collected expression profiles contained 444 samples, which mainly consisted of five biofluid types (blood, urine, stool, saliva and bile) for downstream analysis. The transcriptome profiles of 53 human tissues were obtained from the Genotype-Tissue Expression (GTEx) dataset [10] for analyzing biofluid–tissue gene expression similarity. In addition, to study the difference in biofluid–tissue similarity between sequencing methods, we included the sequencing results of whole component (total RNA), extracellular RNA (exRNA), extracellular vesicle (EV) RNA and stool-derived eukaryotic RNA (seRNA). In practice, the biofluid–tissue similarity index (BTSI) was estimated by the Spearman’s correlation between the transcriptomes of the biofluid and the human tissues.

First, we assessed the correlations between the whole transcriptomes of the biofluid and tissue. Generally, the healthy human tissue transcriptome presented globally positive correlations with the various biofluids (Spearman’s correlation, *p*-value < 0.05; Figure 1C). However, the corresponding similarity level may vary, ranging from 0.70 (platelet-poor plasma, *p*-value = 0.00) to 0.13 (stool seRNA, *p*-value = 8.97 × 10^−85^). Overall, the tissue similarity of biofluid total RNA and biofluid EV RNA were relatively higher than other components, with a maximum of 0.70 (platelet-poor plasma) and a minimum of 0.47 (saliva EV, *p*-value = 0.00). Nevertheless, the correlations between various tissues and biofluid exRNA profiles were rather weak, ranging from 0.38 (serum exRNA, *p*-value = 0.00) to 0.17 (bile exRNA, *p*-value = 2.44 × 10^−130^). The lowest BTSI was observed in stool seRNA (Figure 1C), suggesting a loss of the tissue-derived gene expression pattern during the process in which enterocytes were exfoliated from the colon lumen and excreted into the stool [16].

### 3.2. Tendency and Specificity of Biofluid–Tissue Correlation

Next, we investigated if there were any tendencies in the above biofluid–tissue correlations. Whole transcriptome BTSIs between 53 tissue types and 5 main biofluids were calculated (Figure 1C). Urine and urine EV presented relatively stronger correlations with prostate (BTSI = 0.77, *p*-value = 0.00; BTSI = 0.77, *p*-value = 0.00), pancreas (BTSI = 0.76, *p*-value = 0.00; BTSI = 0.76, *p*-value = 0.00), and bladder tissue (BTSI = 0.75, *p*-value = 0.00; BTSI = 0.76, *p*-value = 0.00), suggesting their potential application in relevant diseases. This finding is in line with previous studies, which reported that urine screening had great potential for pancreatic adenocarcinoma risk prediction [17] and prostate cancer diagnosis [18]. Nevertheless, the phenomenon observed in urine exRNA was not exactly the same. A relatively higher BTSI was still found between urine exRNA and prostate tissue (BTSI = 0.18, *p*-value = 6.46 × 10^−168^). Additionally, we calculated the BTSI between prostate tissue and urine sampled from male and female donors separately to exclude potential sex bias. The results showed a high correlation with prostate tissue in the urine of both male (BTSI = 0.19, *p*-value = 8.97 × 10^−68^) and female (BTSI = 0.20, *p*-value = 1.91 × 10^−185^) donors. However, only a weak correlation was observed between the urine exRNA and pancreas (BTSI = 0.18, *p*-value = 2.96 × 10^−157^) or bladder (BTSI = 0.17, *p*-value = 1.13 × 10^−140^). Thus, urine exRNA seemed not to be an optimal choice for broad urogenital system disease biomarker development. In addition, blood samples (serum and plasma) generally showed a stronger correlation with spleen, lung and adipose (Figure 1C). All of these tissues are major participants in cardiovascular diseases and the hematopoiesis bioprocess [19]. The only exception was the plasma exRNA, which only presented higher correlation with the spleen (BTSI = 0.42, *p*-value = 0.00), and thus might not be an appropriate choice for the development of cardiovascular disease biomarkers. Bile exRNA showed a comparatively stronger correlation with the spleen (BTSI = 0.16, *p*-value = 1.11 × 10^−125^). Intriguingly, the saliva EV exhibited a relatively high correlation with esophagus mucosa (BTSI = 0.48, *p*-value = 0.00) and the stomach (BTSI = 0.47, *p*-value = 0.00). At the same time, the stool seRNA presented comparatively strong correlations with the digestive organs, including the transverse colon (BTSI = 0.15, *p*-value = 9.26 × 10^−103^) and the small intestine (BTSI = 0.14, *p*-value = 1.03 × 10^−95^). Accordingly, we postulate that RNAs in saliva EVs might be suitable biomarkers for the screening of upper digestive tract diseases, while the stool seRNA might be a better candidate for lower digestive tract diseases. The above results appear to imply a tendency toward biofluid–tissue correlation. Generally, from the view of whole transcriptome, biofluids show stronger correlations with the tissues that are involved in their production or bioprocess, and the tissues that are anatomically closer to them.

**Figure 2:**
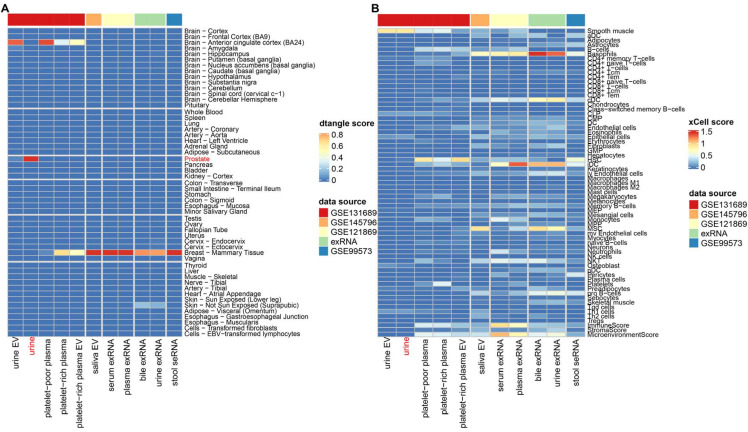
Estimated mixture components of biofluids. (**A**) Heatmap describing the dtangle estimated tissue composition of each biofluid type. (**B**) Heatmap describing the 67 cell-type-specific enrichment scores of each biofluid type.

The dtangle [14] deconvolution method was next used to further explore the contribution of tissues to RNA abundances in various biofluids. Using a reference panel constructed with 53 tissues obtained from the GTEx dataset, the tissue compositions of the biofluids were estimated. Interestingly, a highly specific tissue composition was observed for urine. It seemed that almost all tissue components (99.98%) of urine should be attributed to the prostate (Figure 2A). The same phenomenon was not identified in the urine EV or the urine exRNA, implying a deficiency of this specificity in the cell-free urine component. The gene signature-based method xCell [15] was also used to estimate the cell-type-specific enrichment score of each biofluid. The result showed a high enrichment score for smooth muscle (xCell enrichment score = 0.88), which is the main component of the human prostate gland stroma [20], in the urine sample (Figure 2B). Combined with the high similarity previously observed between the whole transcriptome of the urine and prostate, the urine total RNA seemed to be a promising biomarker source for prostatic diseases.

### 3.3. Biofluid-Derived mRNAs Show Stronger Correlation with Healthy Tissues Than Biofluid-Derived lncRNAs

**Figure 3:**
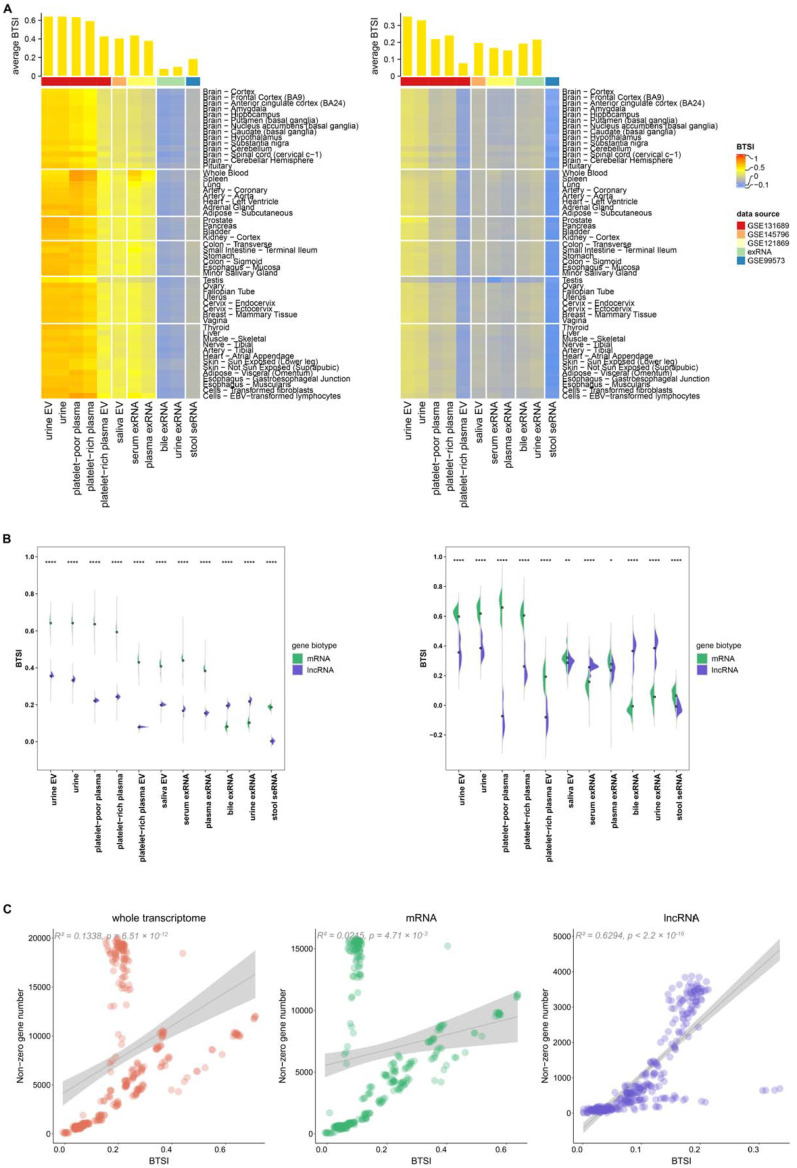
Biofluid–tissue correlation in mRNA and lncRNA view. (**A**) The bar plot at the top and heatmap at the bottom separately describe the BTSI levels between biofluids and all human tissues, as well as each human tissue in mRNA (left) and lncRNA (right). (**B**) The half violin plots show the comparison results of the BTSI levels between BF-mRNAs, T-mRNAs, BF-lncRNAs and T-lncRNAs before (left) and after correction (right); the dot represents the corresponding average BTSI value. ****, ** and * are used to label the *p*-values of the *t*-test results (*p*-value < 0.0001, *p*-value < 0.01, *p*-value < 0.05). (**C**) The scatter plot demonstrates the correlation between BTSI and the non-zero gene number of the biofluid samples in the whole transcriptome (left), mRNA (middle) and lncRNA (right). The *R*^2^ and *p*-values of the linear regression results are labeled.

For a deeper understanding of biofluid–tissue correlations, we next extracted the mRNA and lncRNA transcriptomes separately for BTSI calculation and analysis. In this part of the study, 15,934 mRNAs and 4555 lncRNAs were included. Overall, BF-mRNAs and BF-lncRNAs also presented globally positive correlations with the gene expression pattern in tissues (Figure 3A). The only exception was found in the stool-derived eukaryotic lncRNAs, which showed a non-significant negative correlation with human tissues (BTSI = −0.01, *p*-value = 0.68), suggesting an extreme lack of biofluid–tissue similarity in stool lncRNAs.

As for the biofluid–tissue correlation tendency, a pattern similar to that found in the biofluid whole transcriptome could still be observed for most of the BF-mRNAs and BF-lncRNAs (Figures S1 and S2). However, BF-lncRNAs correlated with T-lncRNAs of different tissues at relatively equal levels, making the tendency not as evident. In particular, for the BF-lncRNAs in the exRNA of serum and plasma, when verified using the above-mentioned *t*-test, an intended correlation with the spleen tissue could not be identified (*t*-test, repeated 10 times, *p*-value > 0.05).

Moreover, BF-mRNAs and T-mRNAs globally present a higher similarity than BF-lncRNAs and T-lncRNAs (*t*-test, *p*-value < 2.2 × 10^−16^; Figure 3B). Only in the bile exRNA and urine exRNA did the BF-lncRNAs demonstrate a higher BTSI (*t*-test, *p*-value < 2.2 × 10^−16^). Considering the result might be affected by non-zero gene number, using 68 as a median expression level in all biofluid samples, we separately filtered out 50 mRNAs and 50 lncRNAs with an average expression value larger than 0 in the samples of each biofluid type for further comparison. After correction, a higher BF-lncRNA T-lncRNA correlation was also found in the serum exRNA (*p*-value = 2.70 × 10^−9^; Figure 3B). As we already know, lncRNAs exert their functions mainly through various direct and indirect means, such as regulating protein activity, genomic targeting and antisense interference [21]. Combined with the above findings, it seemed that the regulation function carried out by BF-lncRNAs are essential for the bile exRNA, urine exRNA and serum exRNA, even though this regulation might be common and has a negligible tissue correlation tendency.

Using linear regression analysis, we also found that BTSI has a significant linear relationship with the non-zero gene number of biofluid samples, in the no-matter-BF whole transcriptome (*R*^2^ = 0.13, *p*-value = 6.51 × 10^−12^), BF-mRNAs (*R* ^2^ = 0.02, *p*-value = 4.71 × 10^−3^), or BF-lncRNAs (*R* ^2^ = 0.63, *p*-value < 2.2 × 10^−16^; Figure 3C). For convenience, this part of the analysis utilized only the RNA-seq profiles of biofluids. This result indicated that higher sequencing depth might facilitate us obtaining more tissue-related information from the biofluid transcriptome. Considering the highest R^2^ found in the lncRNA linear regression result, increasing the sequence depth might benefit a biofluid lncRNA biomarker-related study.

### 3.4. Various Factors Influence Biofluid–Tissue Correlation

**Figure 4:**
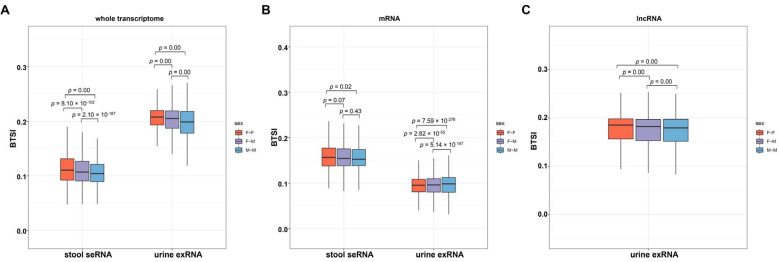
Potential influencing factor of biofluid–tissue correlation. Boxplot depicting the comparison results of the BTSI levels between biofluids and tissues taken from female donors and male donors from the whole transcriptome (**A**), mRNA (**B**) and lncRNA (**C**); the middle line of each box represents the corresponding median BTSI value. The colors of each boxplot indicate biological sexes of biofluid and tissue donors, including both female (F~F), both male (M~M) and different sexes (female biofluid~male tissue or male biofluid~female tissue, F~M). Relevant comparisons were performed with *t*-test, and the resulting *p*-values are provided at the top of each boxplot.

Given the interindividual variation in the human genome and transcriptome [22], we continued to study whether the biological sex of the sample donors might influence the biofluid–tissue correlation. We first extracted the biofluid transcriptome profiles, which provide sex information from both sexes, including urine exRNA profiles (36 female samples and 34 male samples) and stool seRNA profiles (45 female samples and 66 male samples). Then, we separately calculated and compared the BTSIs between biofluid and tissue sampled from both females, both males and different sexes (female biofluid ~ male tissue, and male biofluid ~ female tissue). The results showed a significantly different BTSI between females and males (*t*-test, *p*-value < 0.02; Figure 4), indicating an overall sex bias in biofluid–tissue correlation. In the stool seRNA and the urine exRNA, a significantly higher BTSI was observed in female samples compared to that in male samples.

### 3.5. Function Played by Highly and Lowly Variable Component of Biofluid Transcriptome

**Figure 5:**
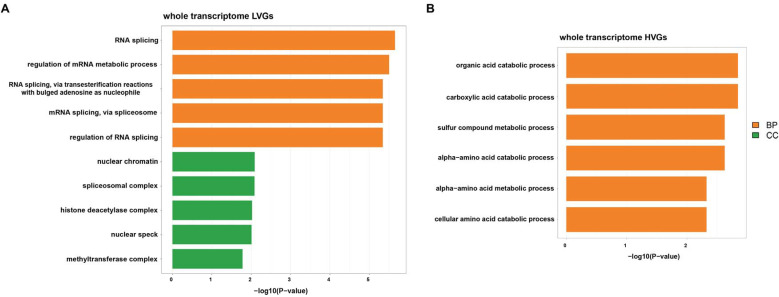
Functional enrichment analysis of highly variable genes (HVGs) and lowly variable genes (LVGs) across biofluid transcriptomes. The bar plots depict functional enrichment results of the top 500 HVGs (**A**) and LVGs (**B**) across the merged biofluid gene expression profiles. The colors of each bar represent different GO items, including the biological process (BP) and cellular component (CC).

Recently, researchers proposed the classification of HVGs and LVGs. The basic assumption is that HVGs (or LVGs) are important components of transcriptomes in which the expression variability may (or may not) be explained by biological heterogeneity among samples [23]. Here, we filtered out the top 500 HVGs as well as the top 500 LVGs across the transcriptomes of various biofluids. Functional enrichment analysis was performed on the identified HVGs and LVGs to explore their biological functions. It seemed that LVGs are enriched in basic biological process-associated pathways, including RNA splicing, protein stability regulation and histone modification (Figure 5A), while HVGs are mostly enriched in pathways relevant to material metabolism, including organic acid catabolism, carboxylic acid catabolism and amino acid metabolism (Figure 5B). Thus, we infer that the material catabolic and metabolic processes differ among various biofluids, while basic biological processes are stable and conserved among various biofluids.

## 4. Discussion

In conclusion, we revealed positive global correlations between various biofluids and human tissues, in no-matter whole transcriptome, mRNA or lncRNA view. In addition, biofluids tend to have stronger correlations with tissues relevant to biofluid production, secretion and transportation, especially with anatomically closer tissues. Most intriguingly, we identified a highly specific correlation between urine and prostate tissue that is supported by both a high BTSI and a high proportion of prostate cells in the urine.

However, significantly stronger correlations were found between BF-mRNAs and T-mRNAs compared to those between BF-lncRNAs and T-lncRNAs. The reverse situation was only observed in bile exRNA, urine exRNA and serum exRNA. Additionally, the biofluid–tissue correlations were presented consistently with respect to whole transcriptomes and mRNAs, but not lncRNAs. Generally, correlations between BF-lncRNAs and different tissues are distributed in a relatively equal fashion.

In addition, our results also suggested the presence of a sex bias in the biofluid–tissue correlations. In the stool seRNA and urine exRNA, the BTSI is higher between the biofluid and the tissue of female donors compared to that of male donors. The above results may help select appropriate biofluid donors for disease biomarker development.

Moreover, we identified the top 500 HVGs and LVGs among the biofluid transcriptomes. The functional enrichment analysis results revealed that the top 500 HVGs are enriched in functions relevant to material catabolism and metabolism, while the top 500 LVGs are enriched in pathways associated with basic biological processes.

In summary, the above findings provide supporting evidence of the potential of various biofluids to be reasonable sources of non-invasive biomarkers. It is also worth noting that factors including the biofluid type, RNA subtype, sequencing depth and donor sex might influence the degree to which we can obtain tissue-associated information from biofluid transcriptomes. We believe a comprehensive evaluation of biofluid–tissue correlations could facilitate the development of biofluid-derived biomarkers for disease diagnosis and investigation.

## Data Availability

The data that support the findings of this study are available from the corresponding author upon reasonable request.

## Supplementary Materials

The following are available online at https://www.mdpi.com/article/10.3390/genes12060935/s1, Figure S1: Tendency in BF-mRNAs T-mRNAs correlation, Figure S2: Tendency in BF-lncRNAs T-lncRNAs correlation, Table S1: Data source of collected biofluid transcriptome profile.
