# Supplementary material for: Comprehensive Analysis of RNA Expression Correlations between Biofluids and Human Tissues
Source: Genes (Basel). 2021 Jun 18;12(6):935. doi: 10.3390/genes12060935 (PMC8234006; doi:10.3390/genes12060935)
Supplement: Supplementary file 1 [file genes-12-00935-s001.zip › genes-1208730-supplementary.pdf]

Supplementary materials

Comprehensive analysis of RNA expression correlations between biofluids and human tissues

Ruya Sun[1], Chunmei Cui[1], Yuan Zhou[1], Qinghua Cui[1 *]

[1]Department of Biomedical Informatics, School of Basic Medical Sciences, Key Laboratory of Molecular Cardiovascular Sciences of the Ministry of Education, Center for Non-coding RNA Medicine, Peking University Health Science Center Beijing, China

Correspondence to:

Qinghua Cui, PhD, Professor

cuiqinghua@hsc.pku.edu.cn

Table S1. Data source of collected biofluid transcriptome profile

Data source	Biofluid type
exRNA atlas	urine exRNA (n = 70)<br>bile exRNA (n = 30)
GSE121869	serum exRNA (n = 54)<br>plasma exRNA (n = 143)
GSE99573	stool seRNA (n = 111)
GSE131689	urine total RNA (n = 2)<br>urine EV RNA (n = 2)<br>platelet-rich plasma total RNA (n = 6)<br>platelet-poor plasma total RNA (n = 14)<br>platelet-rich plasma EV RNA (n = 2)
GSE145796	saliva EV RNA (n = 10)

EV RNA: extracellular vesicle RNA; exRNA: extracellular RNA; seRNA: stool-derived eukaryotic RNA

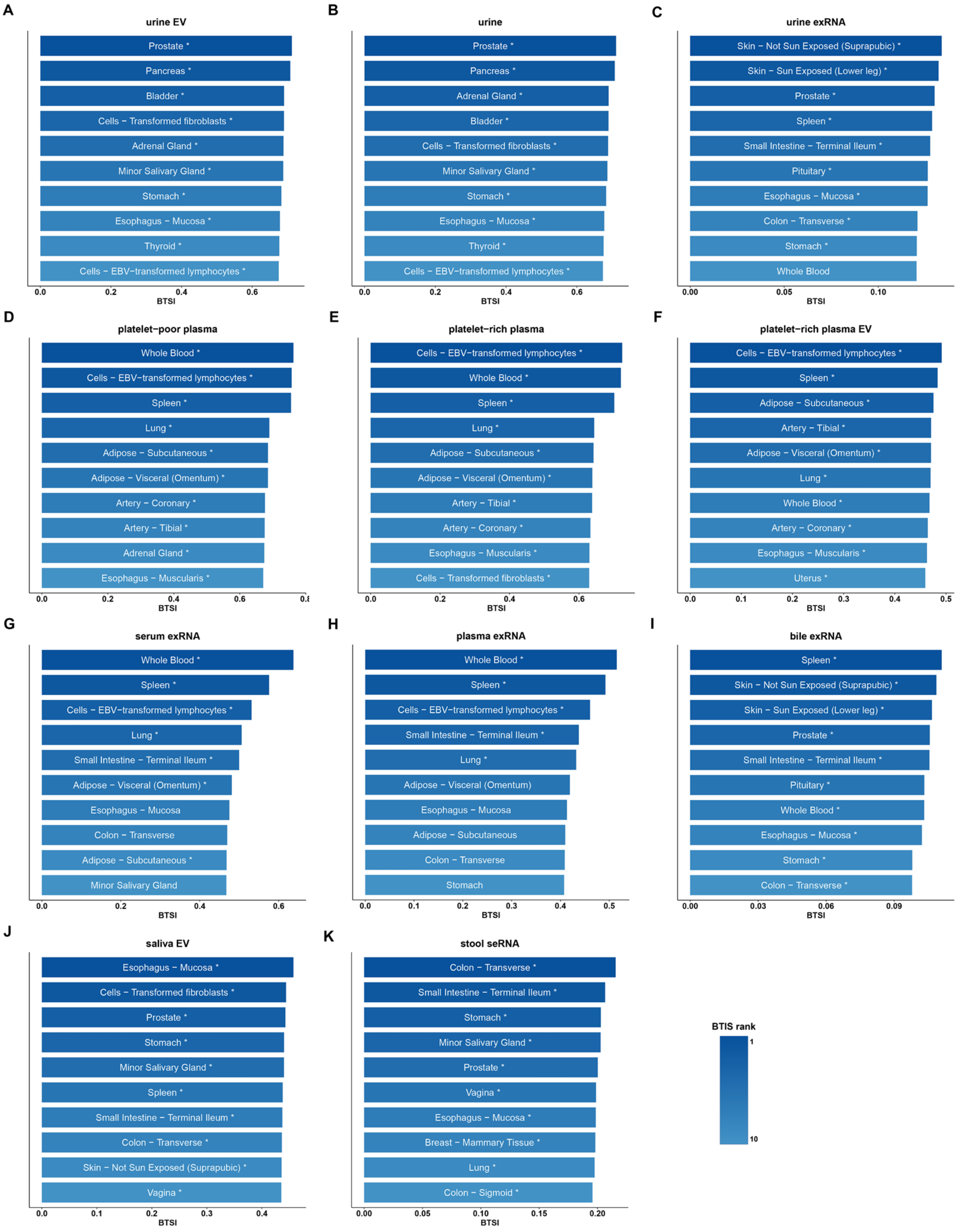

FIGURE S1. Tendency in BF-mRNAs T-mRNAs correlation. The bar plot shows the tissues of the top 10 highest BTSI value for each biofluid type from mRNA view. Only tissues significantly correlated with biofluid (Spearman's correlation, P-value < 0.05) and presented higher correlation with biofluid samples than randomized GTEx samples (n = 1000; t-test, 10 times repeat, P-value < 0.05) are labeled using '*'. The color of each bar represents the rank of the tissue BTSI value for the corresponding biofluid.

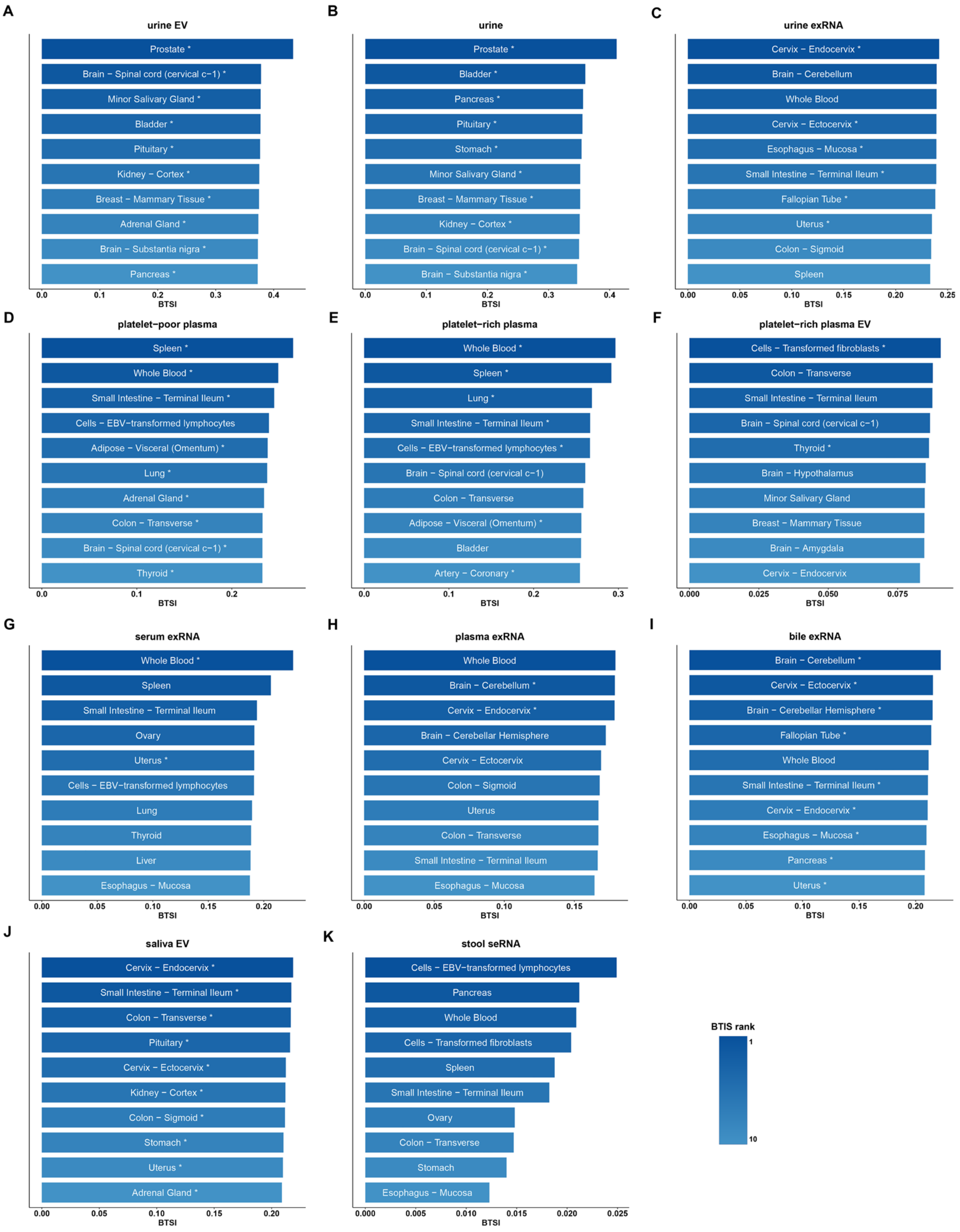

FIGURE S2. Tendency in BF-lncRNAs T-lncRNAs correlation. The bar plot shows the tissues of the top 10 highest BTSI value for each biofluid type from lncRNA view. Only tissues significantly correlated with biofluid (Spearman's correlation, P-value < 0.05) and presented higher correlation with biofluid samples than randomized GTEx samples (n = 1000; t-test, 10 times repeat, P-value < 0.05) are labeled using '*'. The color of each bar represents the rank of the tissue BTSI value for the corresponding biofluid.
